# Human granzyme B binds *Plasmodium falciparum* Hsp70-x and mediates antiplasmodial activity in vitro

**DOI:** 10.1007/s12192-023-01339-8

**Published:** 2023-04-19

**Authors:** Lebogang Ramatsui, Tendamudzimu Harmfree Dongola, Tawanda Zininga, Gabriele Multhoff, Addmore Shonhai

**Affiliations:** 1 Department of Biochemistry and Microbiology, Faculty of Science, University of Venda, Engineering & Agriculture, Thohoyandou, 0950 Limpopo South Africa; 2 Department of Biochemistry, Stellenbosch University, Stellenbosch, 7600 South Africa; 3 Klinik Und Poliklinik Für Strahlentherapie Und Radiologische Onkologie, Klinikum Rechts Der Isar and Central Institute for Translational Cancer Research TU München, TranslaTUM) Einsteinstr. 25, 81675 Munich, Germany

**Keywords:** Malaria, *Plasmodium falciparum*, Heat shock proteins, PfHsp70-x, Granzyme B

## Abstract

Cell surface-bound human Hsp70 (hHsp70) sensitises tumour cells to the cytolytic attack of natural killer (NK) cells through the mediation of apoptosis-inducing serine protease, granzyme B (GrB). hHsp70 is thought to recruit NK cells to the immunological synapse via the extracellularly exposed 14 amino acid sequence, TKDNNLLGRFELSG, known as the TKD motif of Hsp70. *Plasmodium falciparum*-infected red blood cells (RBCs) habour both hHsp70 and an exported parasite Hsp70 termed PfHsp70-x. Both PfHsp70-x and hHsp70 share conserved TKD motifs. The role of PfHsp70-x in facilitating GrB uptake in malaria parasite-infected RBCs remains unknown, but hHsp70 enables a perforin-independent uptake of GrB into tumour cells. In the current study, we comparatively investigated the direct binding of GrB to either PfHsp70-x or hHsp70 in vitro. Using ELISA, slot blot assay and surface plasmon resonance (SPR) analysis, we demonstrated a direct interaction of GrB with hHsp70 and PfHsp70-x. SPR analysis revealed a higher affinity of GrB for PfHsp70-x than hHsp70. In addition, we established that the TKD motif of PfHsp70-x directly interacts with GrB. The data further suggest that the C-terminal EEVN motif of PfHsp70-x augments the affinity of PfHsp70-x for GrB but is not a prerequisite for the binding. A potent antiplasmodial activity (IC_50_ of 0.5 µM) of GrB could be demonstrated. These findings suggest that the uptake of GrB by parasite-infected RBCs might be mediated by both hHsp70 and PfHsp70-x. The combined activity of both proteins could account for the antiplasmodial activity of GrB at the blood stage.

**Supplementary Information:**

The online version contains supplementary material available at 10.1007/s12192-023-01339-8.

## Introduction

Malaria continues to be a global public health burden, with 619,000 deaths recorded in 2021 (WHO 2022). The disease affects mostly children under 5 years who account for more than two-thirds of global malaria deaths (WHO 2022). The persistence of malaria is partly due to the emergence of antimalarial drug-resistant parasites (Wicht et al. 2020). Malaria is caused by members of the *Plasmodium* genus, of which *P. falciparum* causes the most lethal form of the disease.

One of the hallmarks of the parasite is its capability to survive under physiologically distinct environmental conditions as its life cycle transcends across the cold-blooded mosquito vector and the warm-blooded human host. The survival of the parasite across various physiological conditions is partly enabled by the expression of heat shock proteins (Hsps; reviewed by Daniyan et al. 2019). Hsps are protein-folding machineries. Hsps of the parasite do not only provide cytoprotection to the parasite, as several of them are exported into the infected RBCs. In addition, some of them are thought to facilitate the export of several hundreds of parasite proteins to the infected host erythrocytes (reviewed by Jonsdottir et al. 2021). In this respect, these parasite Hsps are directly or indirectly involved in host cell modifications. Consequently, it is assumed that the expression of the parasite Hsps correlates with clinical malaria progression (Pallavi et al. 2010). In addition, the upregulated expression of parasite Hsps is linked to antimalarial drug resistance (Rocamora et al. 2018). For this reason, antiplasmodial agents that target the parasite Hsps have been proposed as possible effective interventions against malaria (Shonhai 2010; Muthelo et al. 2022) .

The Hsp70 family members are molecular chaperones that facilitate the folding of newly synthesised proteins, refolding of misfolded proteins, disaggregation of protein aggregates and membrane translocation of sub-organelle-bound and secretory proteins (Hartl 1996; Mayer and Bukau 2005; Radons 2016). Hsp70s structurally consists of two major domains, the 45 kDa N-terminal ATPase domain (NBD) and the 25 kDa C-terminal substrate-binding domain (SBD) (Flaherty et al. 1990; Wang et al. 1993). The Hsp70-SBD is further sub-divided into a β-domain, consisting of the substrate-binding pocket, and an α-helical lid (Wang et al. 1993). The SBD and the ATPase are joined by a flexible linker region that facilitates inter-domain communications (reviewed by Chakafana et al. 2019).

*P. falciparum* expresses six different Hsp70s that are localised in different cellular compartments of the parasite (Shonhai 2021). Among these, only PfHsp70-x is exported to the cytosol of the infected RBCs (Külzer et al. 2012). PfHsp70-x is thought to associate with *P. falciparum* erythrocyte membrane protein 1 (PfEMP1) (Charnaud et al. 2017). This suggests that PfHsp70-x is involved in modulating the integrity of the infected erythrocyte.

Due to the growing threat of antimalarial drug resistance, the search for safer and more reliable antimalarial therapies remains an important topic. Granzyme B (GrB) is a serine protease that is found in the granules of cytotoxic T cells and natural killer (NK) cells. It has been proposed that GrB possesses antiplasmodial activity (Böttger et al. 2012; Kapelski et al. 2015). In addition, GrB that was attached to a signalling peptide showed an improved delivery to the parasite-infected RBCs and was reported to possess antimalarial activity at nanomolar concentrations (Kapelski et al. 2015). Therefore, it meets the criteria for prospective antimalarial drugs as per the guidelines of Medicines for Malaria Venture (MMV; https://www.mmv.org/).

It has been proposed that GrB enters a malaria parasite-infected erythrocyte through its interaction with Hsp70 which resides on the surface of the erythrocyte (Böttger et al. 2012). In line with these findings, a previous study proposed that Hsp70 that is exposed on the cell surface of tumour cells facilitates their recognition and targeting by NK cells (Gross et al. 2003). It is assumed that a 14-mer sequence, the so-called TKD motif (TKDNNLLGRFELSG; amino acids 450–463) of the C-terminal domain of human Hsp70 (hHsp70), facilitates activation of NK cells, recruitment and uptake of GrB into tumour cells habouring Hsp70 on their cell surface (Sutton et al. 2003; Gross et al. 2003). However, the role of Hsp70 in GrB-mediated antiplasmodial responses remains to be elucidated. While erythrocytes are incapable of protein synthesis, they host Hsp70 of human origin. Therefore, *P. falciparum*-infected erythrocytes habour both hHsp70 and PfHsp70-x (reviewed in Zininga et al. 2021). It remains unknown which of the two Hsp70s facilitates the uptake of GrB. The current study explored the capabilities of both hHsp70 and PfHsp70-x to interact with GrB in vitro. We discuss the implications of our findings with respect to the uptake of GrB via cell surface-bound Hsp70 by malaria parasite-infected RBCs.

## Methods and materials

### Materials

Unless otherwise stated, the reagents used in this study were procured from Merck (Darmstadt, Germany), Sigma-Aldrich (Darmstadt, Germany) and Thermo Scientific (IL, USA). The plasmid constructs encoding for the full-length PfHsp70-x_F_ (denoted as pQE30/PfHsp70-x_F_), the truncated version of the protein with the C-terminal EEVD missing (denoted as pQE30/PfHsp70-x_T_) and human Hsp70 (denoted as pQE30/hHsp70) were previously described (Mabate et al. 2018). The following antibodies were used in this study: α-PfHsp70-x (Mabate et al. 2018), α-His antibodies (Sigma-Aldrich, USA) and α-GrB antibodies (Sigma-Aldrich, USA). Granzyme B used in the study was obtained from Hölzel Diagnostics, Cologne, Germany.

### Multiple sequence alignment

Multiple sequence alignments of PfHsp70-x (PF3D7_0831700.1) and its truncated version lacking the C-terminal EEVN residues and hHsp70 (NP_005336.3) were conducted using BioEdit software (https://bioedit.software.informer.com/7.2/).

### Recombinant protein production

The recombinant proteins were heterologously produced using a previously described protocol (Mabate et al. 2018) with minor modifications. Briefly, plasmids (pQE30/PfHsp70-x_F_, pQE30/PfHsp70-x_T_ and pQE30/hHsp70) were separately transformed into *E. coli* BL21 cells. The recombinant proteins were overexpressed in terrific broth (TB) (1.2% tryptone, 2.4% yeast, 0.5% glycerol, 17 mM KH_2_PO_4_, 72 mM K_2_HPO_4_ supplemented with 100 μg/mL ampicillin) at 37 °C and induced using 1 mM isopropyl-β-D-1-thiogalactopyranoside (IPTG). The cells were harvested by centrifugation at 15,000 g for 1 h. The pellets were then re-suspended in 10 mL lysis buffer (10 mM Tris–HCl, pH 7.5, 300 mM NaCl and 10 mM imidazole, containing 1 mM EDTA, 1 mM phenylmethylsulfonyl fluoride (PMSF) and 1 mg/mL lysozyme). The whole *E. coli* lysate was frozen at − 80 °C freezer and was thawed on ice. Polyethyleneimine (PEI) at 0.1% (*v*/*v*) was added to the lysate to solubilise the proteins and precipitate nucleic acids. The lysate was centrifuged at 5000 g for 20 min at 4 °C. The supernatant was loaded onto HisPur™ nickel-charged nitrilotriacetic acid (Ni–NTA) immobilised metal affinity chromatography (IMAC) (Thermo Scientific, USA) and incubated at 4 °C for 4 h. The column was washed with wash buffer (lysis buffer supplemented with 25 mM imidazole) and protein eluted using elution buffer (lysis buffer supplemented with 500 mM imidazole). The expression and purification of recombinant were analysed with SDS-PAGE and western blot analysis using the α-PfHsp70-x (Mabate et al. 2018) and α-His antibodies (Sigma-Aldrich, USA).

### Investigation of the secondary structure and stability of the Hsp70 proteins

The secondary structures of recombinant hHsp70, PfHsp70-x_F_ and PfHsp70-x_T_ were analysed by Far-UV circular dichroism using a J-1500 CD spectrometer (JASCO Ltd., Japan) as previously described (Mabate et al. 2018). Briefly, recombinant proteins (at 0.5 μM) were suspended in PBS (137 mM NaCl, 27 mM KCl, 4.3 mM Na_2_HPO_4_ and 1.4 mM KH_2_PO_4_, at pH 7.4) and analysed using a 0.1 cm path-length quartz cuvette (Hellma). The spectra were averaged for 7 scans after baseline correction (subtraction of spectrum from the buffer in which the proteins were excluded). Secondary structure predictions were conducted as previously described (Zininga et al. 2015). The spectra were deconvoluted to α-helix, β-sheet, β-turn and unordered regions using the DichroWeb server (CDSSTR reference database) (http://dichroweb.cryst.bbk.ac.uk) (Sreerama and Woody 2000; Whitmore and Wallace 2008). The folded fraction ratio was determined by comparing the molar residue ellipticity at 222 nm of native protein at 20 °C and at variable temperatures.

### Assessment of the secondary and tertiary structures of Hsp70s

Intrinsic fluorescence for all three proteins (PfHsp70-x_F_, PfHsp70-x_T_ and hHsp70) was analysed to infer the tertiary conformation of the proteins as previously described (Mabate et al. 2018). To investigate the effect of a denaturant (urea) on the integrity of the proteins, each of the recombinant proteins (at 2 μM) was incubated in PBS for 20 min at 20 °C in the presence of variable concentrations (0 M–8 M) of urea. Fluorescence spectra were recorded between 300 and 400 nm after initial excitation at 295 nm using a JASCO FP-8200 spectrofluorometer (Tokyo, Japan).

### Analysis of chaperone activity of Hsp70

The chaperone function of PfHsp70-x_F_, PfHsp70-x_T_ and hHsp70 was investigated by monitoring their ability to suppress heat-induced aggregation of a model substrate, malate dehydrogenase (MDH) from the *porcine* heart (Sigma-Aldrich, USA) as previously described (Shonhai et al. 2008; Zininga et al. 2016). Briefly, the capability of each protein to suppress thermally induced aggregation of MDH was monitored spectrophotometrically. MDH (0.6 μM) was added to the preheated buffer (50 mM Tris, pH 7.4, 100 mM NaCl) at 51 °C. The temperature was maintained at 51 °C for 80 min, and the absorbance was monitored at 340 nm in 5-min intervals using a SpectraMax M3 spectrometer (Molecular Devices, USA). BSA was used as a non-chaperone control in this assay. The data were analysed using GraphPad Prism 9.0 software (GraphPad Software, CA, USA).

### Investigation of the interaction of Hsp70 with granzyme B

#### Slot blot assay

The interaction of PfHsp70-x/hHsp70 with GrB was explored using slot blot following the protocol previously described (Zininga et al. 2016). Briefly, varying concentrations (5 μg, 10 μg and 20 μg) of recombinant PfHsp70-x_F_ and hHsp70 were immobilised onto nitrocellulose membrane using the Bio-Dot ^TM^SF (Bio-Rad, USA) apparatus. As controls, 20 μg of bovine serum albumin (BSA), PfHsp70-x_T_ and human GrB were immobilised onto the membrane. The membrane was then blocked with 5% fat-free milk prepared in Tris buffer saline (TBS, 50 mM Tris, 150 mM NaCl, pH 7.5) for 1 h at 25 °C, followed by washing with TBST (TBS supplemented with 1% Tween 20) three times for 15 min. The interaction of GrB with a synthetic TKD peptide (TKDNNLLGKFQLEG; produced by Genscript, Piscataway, NJ, USA) representing the 14-mer TKD motif of PfHsp70-x was explored using slot blot analysis. The peptide was immobilised onto the nitrocellulose membrane in varying concentrations (5 μg, 10 μg and 20 μg). A second peptide, NGL, constituted by residues, NGLTLKNDFSRLEG, representing a previously described (Stangl et al. 2011) scrambled sequence of the TKD motif of hHsp70, was used as a negative control. Subsequently, the immobilised proteins/peptides were overlaid with GrB (10 μg/mL) prepared in 5% fat-free milk and incubated overnight at 4 °C. After the washing steps, the membranes were incubated with the mouse-raised monoclonal α-GrB primary antibody [1:2000] (Sigma-Aldrich, USA) followed by incubation with goat-raised α-mouse IgG secondary HRP-conjugated antibody [1:4000] (Sigma-Aldrich, USA). The immunoblots were visualised using a chemiluminescent substrate (ECL) (Thermo Scientific, USA) as per the manufacturer’s instructions. The images were captured using the ChemiDoc Imaging System (Bio-Rad, USA). The densitometric analysis was conducted using the Image Lab™ software (Bio-Rad, USA).

#### Enzyme-linked immunosorbent assay (ELISA)

ELISA was used to explore the direct interaction of GrB and Hsp70 using a previously described protocol (Biesiadecki and Jin 2011; Mabate et al. 2018). Briefly, recombinant proteins (5 μg) PfHsp70-x_F_, PfHsp70-x_T_, hHsp70 and BSA suspended in 5 mM NaHCO_3_ pH 9.5 were immobilised on a 96-well microtiter plate by passive adsorption and incubated overnight at 4 °C. After incubation, the plate was washed to remove unbound protein with TBST (TBS supplemented with 0.1% Tween 20). The wells were then blocked with 150 μL of 5% fat-free milk prepared in TBS and incubated at 25 °C for 1 h. The wells were then washed using TBST three times for 10 min. Serial dilutions of the analyte GrB (0–1000 nM) were prepared in 2.5% fat-free milk prepared in TBS, and 100 μL of each dilution was added into the wells and incubated at 25 °C for 2 h. The wells were then washed three times before the addition of monoclonal α-GrB primary antibody (1:2000) to each well and incubated at 25 °C for 1 h. The secondary goat-raised HRP-conjugated α-mouse antibody (1:2000) was then added to the well and incubated for 1 h at 25 °C. The excess unbound antibody was washed three times for 10 min using buffer A. The bound secondary antibody was monitored by the addition of the substrate 3,3′,5,5′-tetramethylbenzidine (TMB) (Cambridge Bioscience, UK). The SpectraMax M3 microplate reader (Molecular Devices, USA) was used to monitor the colour development over 5-min intervals for 30 min at 370 nm. The resulting absorbance values were plotted against time. Furthermore, to investigate the effects of nucleotides on the interaction, the assay was repeated in the presence of 5 mM ATP/ADP.

Baseline subtraction for readings obtained from uncoated and BSA negative control wells were factored as part of the data analysis. Readings obtained at the highest concentrations of GrB were averaged to represent maximum (100%) binding. GraphPad Prism 9.0 (GraphPad Software, CA, USA) was used to plot a titration curve against a log scale, and the curve was fitted to determine the equilibrium binding constant (Kd). The Kd score represents the concentration of GrB required to reach 50% maximal binding.

#### Surface plasmon resonance (SPR) spectroscopic analysis

To further validate the association of GrB with PfHsp70-x/hHsp70, surface plasmon resonance (SPR) spectroscopy was conducted. The analysis was performed at 25 °C using the BioNavis Multi Parametric (MP-SPR) Navi 420A ILVES system (BioNavis, Finland). As ligands, the respective recombinant proteins (PfHsp70-x/hHsp70) were each immobilised onto gold-functionalised three-dimensional carboxymethyl dextran sensor (CMD 3D 500L chips at concentrations of 100 µg/mL as previously described (Chakafana et al. 2021). A PfHsp70-x_T_ and GrB (control) were also immobilised onto a separate chip under the same conditions. The sensor chips were stabilised by injecting the running buffer [PBS-Tween 20 (4.3 mM Na_2_HPO_4_, 1.4 mM KH_2_PO_4_, 137 mM NaCl, 3 mM KCl, 0.005% (*v*/*v*) Tween 20 and 20 mM EDTA; pH 7.4)] as previously described (Zininga et al. 2016; Mabate et al. 2018). Following stabilisation, as the analyte, GrB was then injected in series as aliquots of 0, 125, 250, 500, 1000 and 2000 nM and a flow rate of 25 μL/min into each horizontal flow channel. The assay was repeated by immobilising GrB on the chip surface and injecting the TKD/NGL peptides as analytes at concentrations of 0, 125, 500, 750 and 1000 nM. Association between ligand and analyte was allowed for 8 min to reach steady-state and dissociation was monitored for a total of 5 min. Steady-state kinetic data were processed and analysed using TraceDrawer software version 1.8 (Ridgeview Instruments, Sweden). Kinetic evaluations were conducted on the sensorgrams after 1:1 global fitting using the Langmuir model for all 5 concentrations. Statistical analyses were conducted using a one-way ANOVA (*p* < 0.01).

### Investigation of the antiplasmodial activity of granzyme B

*P. falciparum* 3D7 parasites were maintained in continuous culture as previously described (Trager and Jensen 1976; Zininga et al. 2015). The production of lactate dehydrogenase (pLDH) by *P. falciparum* 3D7 cells maintained at the blood stage was used to monitor parasite viability in the presence of varying amounts (0–25 μg/mL) of GrB, as previously described (Zininga et al. 2017). A preliminary parasite growth assay was conducted using 25 μg/mL of GrB. The IC_50_ of GrB was determined at a concentration range of 0.001–20 μg/mL. Analysis of the pLDH data for the growth inhibition assay was carried out using GraphPad Prism 9 (GraphPad Prism software, CA, USA). The assay was conducted in triplicate for 3 different experiments (*n* = 3).

## Results

### Analysis of the TKD motifs of PfHsp70-x and hHsp70

**Fig. 1:**
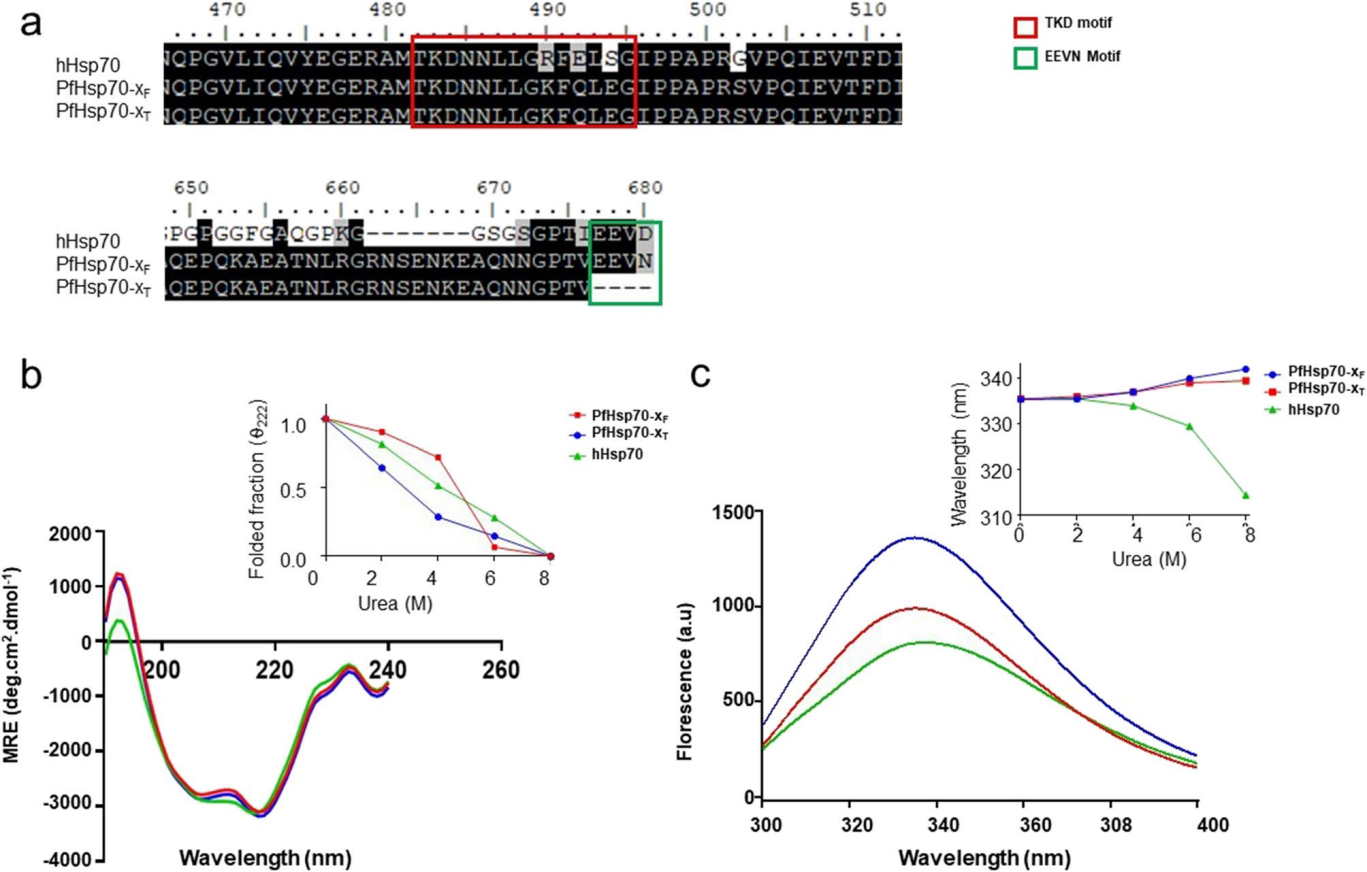
Analysis of the primary, secondary and tertiary structure of Hsp70 proteins. Multiple sequence alignments of PfHsp70-x_F_, PfHsp70-x_T_ and hHsp70 were conducted using BioEdit software (https://bioedit.software.informer.com/7.2/) (**a**). The residue positions in the alignment are based on the PfHsp70-x sequence. CD spectroscopic analysis was conducted on the proteins (**b**). The CD spectroscopic analysis was repeated in the presence of varying concentrations of urea and the resultant folded fractions of each protein were estimated (**b** insert). The intrinsic fluorescence analysis was conducted on each protein in the absence of urea (**c**). Tryptophan fluorescence emission spectra were recorded in the presence of varying levels of urea and the estimated folded fractions of the proteins were presented (**c** insert)

Sequence alignment demonstrated that the TKD motif of Hsp70 is conserved in both PfHsp70-x and hHsp70 (Fig. 1a). However, we observed a substitution at the position, 458R of hHsp70 to 489 K in PfHsp70-x. As both residues possess positively charged side chains, this is a conservative substitution. While hHsp70 is represented by the negatively charged glutamate (E) residue at position 460, PfHsp70-x is represented by the polar uncharged glutamine (Q) residue at position 491. A non-conservative substitution is observed in position 462S (polar, uncharged) at the C-terminus of the TKD motif of hHsp70 versus 493E (negatively charged) of PfHsp70-x.

### Comparative analysis of the secondary and tertiary structural orientations of PfHsp70-x and hHsp70

To further explore the structural features of the Hsp70 proteins in humans and *Plasmodium*, we employed CD and intrinsic fluorescence spectrometry. To facilitate the analyses, recombinant forms of the proteins were expressed and purified (Supplementary Figure S1). The secondary far UV CD spectra of PfHsp70-x (both full-length and truncated versions) and hHsp70 exhibited two minima (troughs) at 209 and 221 nm, respectively (Fig. 1b). This spectrum confirms the α-helical character of the proteins. The relative stabilities of the proteins were compared after the addition of the chaotropic agent urea by monitoring their respective secondary structures using CD spectroscopy at 221 nm. As expected, with increasing concentrations of urea, the proteins lost their secondary structural folding. However, PfHsp70-x_F_ exhibited a higher stability than hHsp70 (Fig. 1b). Furthermore, PfHsp70-x_T_ was less stable than the full-length PfHsp70-x_F_ protein, confirming the importance of the C-terminal EEVN motif in conferring stability to this protein, as previously reported (Mabate et al. 2018).

In a separate experiment, the tertiary structures of PfHsp70-x_F_, PfHsp70-x_T_ and hHsp70 were evaluated using intrinsic tryptophan-based fluorescence analysis (Fig. 1c). As expected, increasing concentrations of urea led to a decrease in tryptophan fluorescence signals due to signal quenching (Fig. 1c). Notably, in response to urea treatment, both PfHsp70-x_F_ and PfHsp70-x_T_ exhibited a red wavelength shift, while hHsp70 exhibited a blue wavelength shift (Fig. 1c). The red shift suggests that both PfHsp70-x_F_ and PfHsp70-x_T_ acquired a more open conformation in the presence of urea. The blue shift observed for hHsp70 could be indicative of an aggregated status of this relatively unstable protein.

### Suppression of heat-induced aggregation of malate dehydrogenase (MDH) by Hsp70

**Fig. 2:**
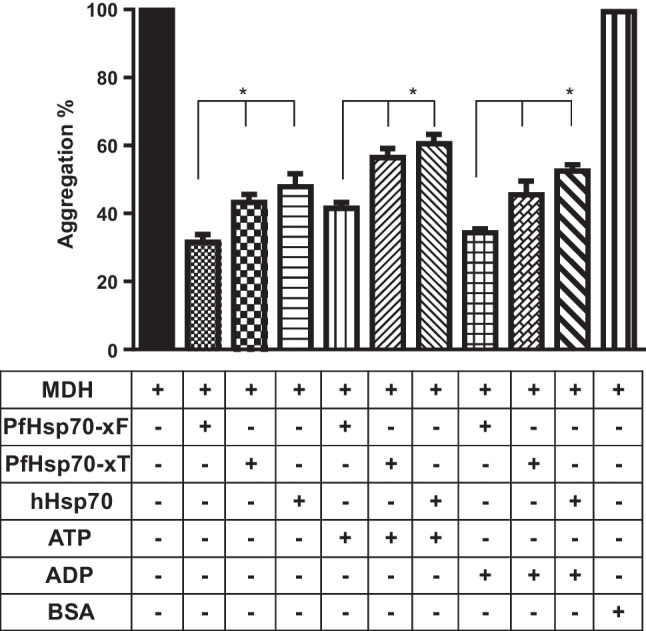
Hsp70s suppress heat-induced aggregation of malate dehydrogenase. The heat-induced aggregation of the model substrate MDH was monitored using a SpectraMax M3 spectrometer (Molecular Devices, USA). The temperature was set at 51 °C, and the assay was performed at 340 nm. The aggregation of MDH in the absence of a chaperone was defined as 100% aggregation as a reference point. The capability of PfHsp70-x_F_, PfHsp70-x_T_ and hHsp70 to suppress the heat-induced aggregation of MDH was monitored either in the presence of ATP/ADP. BSA was used as a non-chaperone control protein. Standard errors shown were obtained from three independent replicate assays. GraphPad Prism 9.0 was used to conduct one-way ANOVA to determine the significance of the differences (*p* < 0.05)*

The capabilities of the three Hsp70 types to suppress heat-induced aggregation of the model substrate MDH were investigated by measuring the change in turbidity of the reaction mixtures at 340 nm. MDH subjected to heat stress in the absence of the chaperones at 51 °C gradually aggregated (Fig. 2). However, in the presence of the chaperones, the aggregation of MDH was found to be suppressed. As previously reported (Mabate et al. 2018), PfHsp70-x_F_ was more effective in suppressing the aggregation of MDH than its truncated version PfHsp70-x_T_ (Fig. 2). In addition, PfHsp70-x_F_ was more effective in suppressing MDH aggregation than hHsp70 (Fig. 2). In agreement with previous findings (Mabate et al. 2018), in the presence of ADP, all three Hsp70s exhibited a higher chaperone activity than in the presence of ATP (Fig. 2). This confirmed the nucleotide-dependency of the chaperone activity.

### Slot blot analysis demonstrates that the TKD motif of PfHsp70-x directly interacts with human GrB

**Fig. 3:**
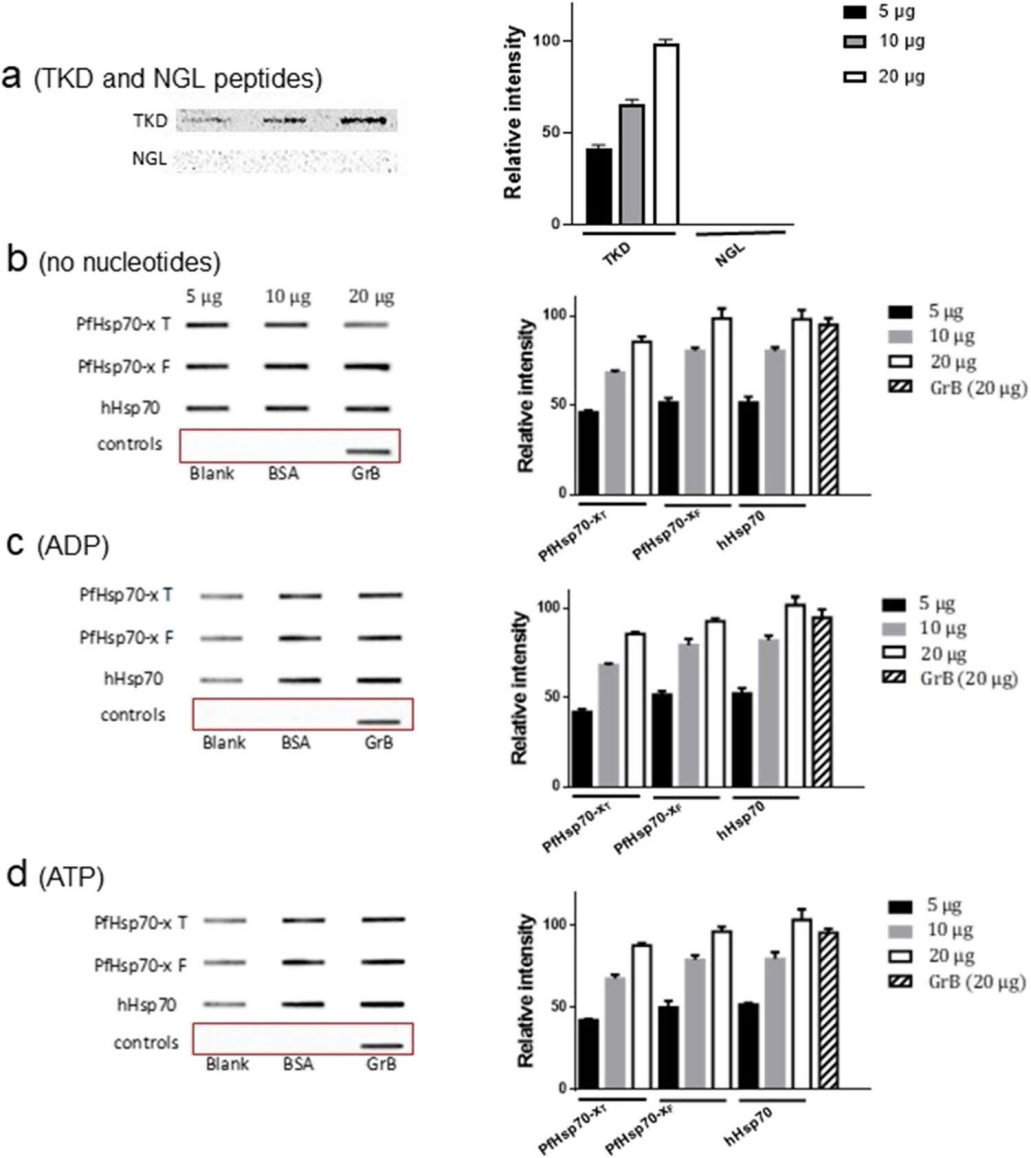
Slot blot demonstrating granzyme B interaction with both PfHsp70-x and hHsp70. Interaction between the immobilised bait peptides, TKD/NGL (**a**) or any of the three Hsp70 proteins (PfHsp70-x_F_, PfHsp70-x_T_ and hHsp70) with the prey protein (human GrB) was probed with α-GrB antibody. The interaction of the three Hsp70s with GrB was investigated either in the absence of nucleotides (**b**) or in the presence of 5 mM ADP (**c**)/ATP (**d**), respectively. The respective densitometric analysis is presented in the accompanying bar graph on the right-hand side of each blot

First, we demonstrated that the synthetic TKD peptide of PfHsp70-x is capable of interacting with GrB (Fig. 3a). On the other hand, the peptide, NGL, representing the negative control did not bind to GrB as previously demonstrated (Stangl et al. 2011). Furthermore, the slot blot analysis demonstrated that GrB interacted with all three Hsp70s in a concentration-dependent manner (Fig. 3). The fact that both PfHsp70-x_F_ and its truncated version, PfHsp70-x_T_, interacted with GrB shows that the C-terminal EEVN motif of PfHsp70-x is not essential for interaction with GrB. Furthermore, the specificity of GrB for Hsp70 was demonstrated by a lack of its interaction with BSA.

### ELISA and SPR analysis validated the direct interaction of PfHsp70-x and its TKD motif with GrB

**Fig. 4:**
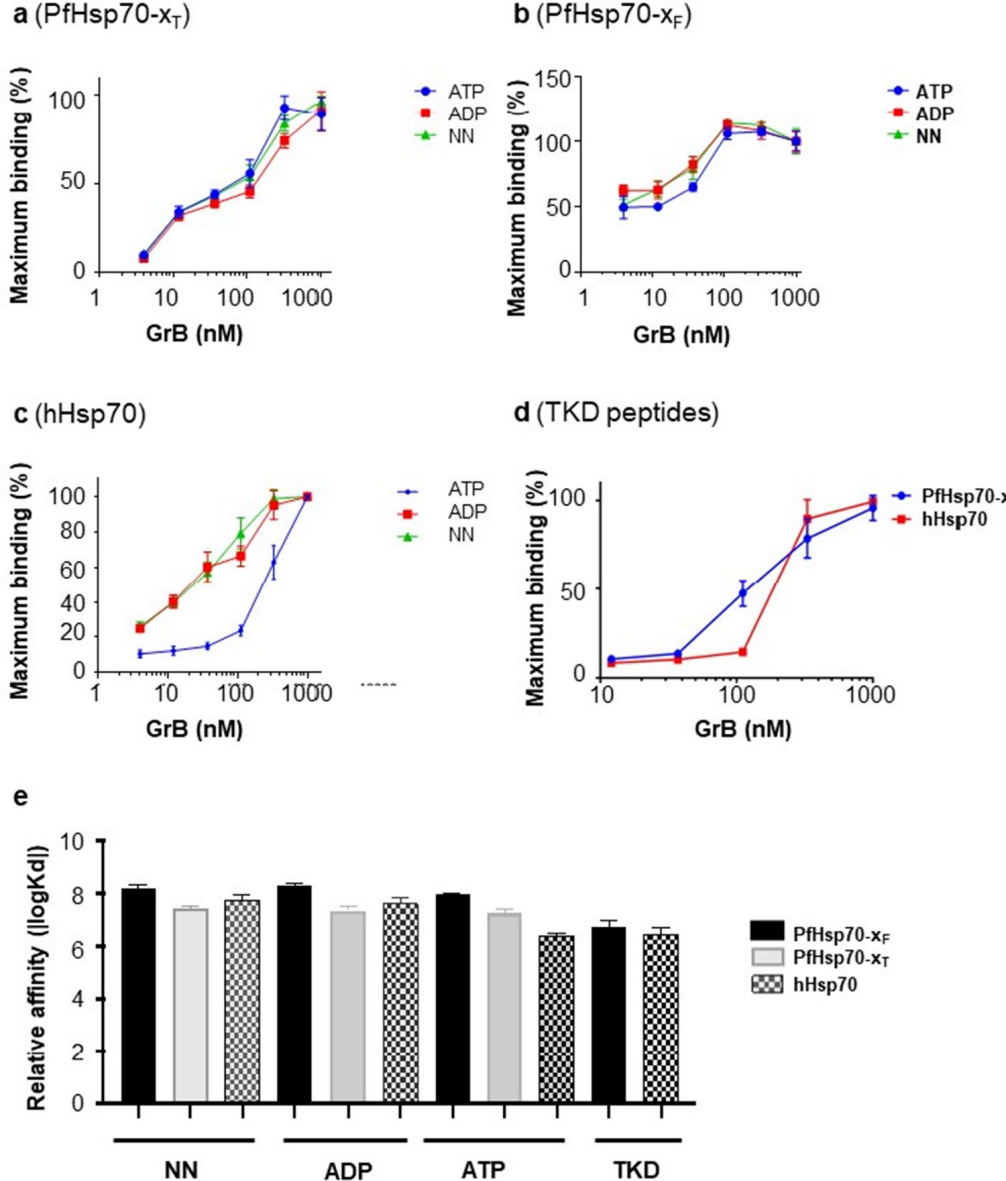
ELISA demonstrated direct interaction of the TKD motifs of PfHsp70-x and hHsp70 with GrB. ELISA was used to generate titration curves against a log scale for the association of human GrB with PfHsp70-x_F_/PfHsp70-x_T_ or hHsp70 (**a–c**). The assay was repeated to explore the interaction of the respective TKD motifs of the Hsp70s with GrB (**d**). The relative affinities of the interaction were plotted as log K_d_ values (**e**). The data represent mean values of 3 independent assays conducted with different protein batches

Since Hsp70 interacts with its folding clients in a nucleotide (ATP/ADP)-dependent manner, we compared the binding of the various Hsp70s to GrB in the absence of nucleotide and in the presence of either ADP or ATP. A concentration-dependent interaction of the different Hsp70s with GrB was shown by ELISA (Supplementary Figure S2). There was no evidence that PfHsp70-x_F_ or its derivative, PfHsp70-x_T_, interacted with GrB in a nucleotide-dependent fashion (Fig. 4a, b). However, in the presence of ATP, we observed a drop in the affinity of hHsp70 to GrB (Fig. 4c). To address the question of whether this difference is due to an artefact or reflects that Hhsp70 and PfHsp70-x may be uniquely regulated by ATP with respect to their interaction with GrB, we investigated whether the TKD motifs of the two Hsp70 proteins interact with GrB differently. Since the 14-mer TKD motifs of PfHsp70-x and hHsp70 share three amino acid substitutions, we investigated the direct interaction of the respective TKD motifs with GrB by ELISA (Fig. 4d, e) and SPR analysis. The ELISA data demonstrated a concentration-dependent interaction of GrB with both TKD motifs (Fig. 4d). The estimated Kd values obtained by ELISA revealed that both TKD motifs bind GrB with similar affinities (Fig. 4e). However, because of the limited sensitivity of the ELISA, we employed SPR analysis as a more sensitive approach.

**Fig. 5:**
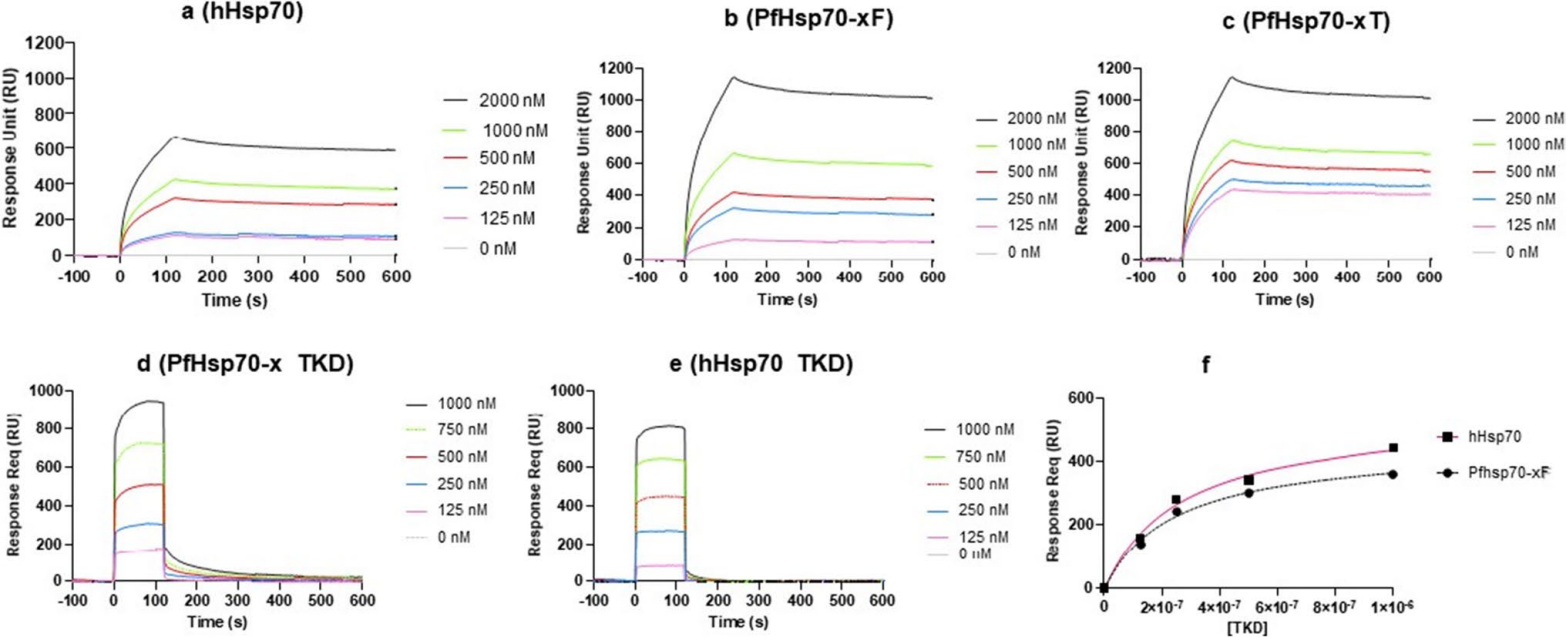
Sensorgrams representing the interaction of GrB with PfHsp70-xF/PfHsp70-x-T/hHsp70 and their respective TKD motifs. The SPR-generated sensorgrams were used to determine the affinity of GrB interaction with hHsp70 (**a**), Pfhsp70-xF (**b**), PfHsp70-xT (**c**), TKD motif of PfHsp70-x (**d**) and TKD motif of hHsp70 (**e**), respectively. The equilibrium constant analysis for the association of GrB with the TKD peptides is shown (**f**)

The SPR data demonstrated that all three proteins interact with GrB, but PfHsp70-x exhibits a much higher affinity for GrB than hHsp70 (Fig. 5; Table 1). Similarly, the TKD motif of PfHsp70-x exhibited a higher affinity for GrB than that of hHsp70. This suggests that the higher affinity for GrB exhibited by full-length PfHsp70-x is mediated in part by its TKD motif. Furthermore, the removal of the C-terminal EEVN motif significantly reduced the affinity of PfHsp70-x for GrB irrespective of the ADP/ATP status. As a control experiment to validate the SPR assay, the interaction of the NGL peptide with GrB was investigated using SPR. As expected (Stangl et al. 2011) no interaction occurred between the peptide, NGL and GrB (data not shown).

**Table 1:** Hsp70-GrB binding kinetic parameters

Ligand	Analyte	Nucleotide	Ka (1/Ms) e^3^	Kd (1/s)	K_D_ (µM)	Chi^2^
PfHsp70-xF	GrB	-	4.35 (± 0.8)	1.64 (± 0.7) e-5	0.004	8.7
		ADP	3.85 (± 6.5)	7.06 (± 0.6) e-6	0.001	9.8
		ATP	6.99 (± 0.96)	2.05 (± 0.9) e-5	0.001	9.7
PfHsp70-xT	GrB	-	2.14 (± 0.15)	7.32 (± 1.1) e-4	2.37	2.0
		ADP	2.56 (± 1.7)	2.24 (± 0.8) e-5	2.82	1.7
		ATP	5.87 (± 0.96)	1.23 (± 0.2) e-4	4.2	1.5
hHsp70	GrB	-	2.37 (± 0.59)	9.89 (± 2.0) e-4	1.53	6.5
		ADP	1.62 (± 0.8)	1.21 (± 0.3) e-3	5.61	9.0
		ATP	3.95 (± 0.83)	8.98 (± 1.7) e-4	0.20	5.9
PfHsp70-x TKD	GrB	-	3.13 (± 0.77)	3.62 (± 0.4) e-5	0.020	1.9
hHsp70	GrB	-	2.93 (± 0.4)	2.17 (± 0.2) e-5	0.004	1.7

Legend: *ka* represents the association rate constants of the respective ligands and analytes, whilst *kd* represents the dissociation rate constants. The K_D_ values represent the relative affinity between the ligand and analyte, and the Chi^2^ value shows the goodness of fit of the data

Based on the K_D_ values obtained for both PfHsp70-x_F_ and PfHsp70-x_T_, nucleotides appeared not to influence the association of Hsp70 and GrB (Table 1). However, the K_D_ values obtained by SPR analysis suggest that hHsp70 exhibited a reduced affinity for GrB in the presence of ATP. It is therefore plausible that while ATP may not influence the association of PfHsp70-x with GrB, the interaction of hHsp70 with GrB might be affected by ATP.

### Granzyme B exhibits antiplasmodial activity

**Fig. 6:**
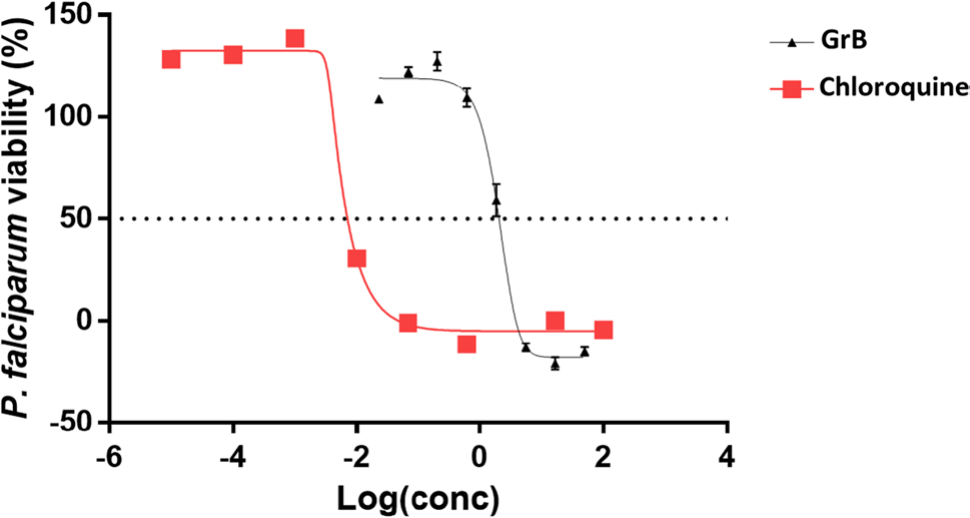
The in vitro susceptibility of *P. falciparum* to GrB is represented as a normalised dose–response curve. The curves represent data obtained from assays conducted in the presence of chloroquine (IC_50_ of 0.0085 μM) or GrB (IC_50_ of 0.546 μM), respectively. IC_50_ values were calculated from the generated dose–response curves obtained by GraphPad Prism 9 analysis. The experiment was performed in triplicates with biological repeats. Standard errors of the mean were obtained from three independent assays

The ability of human GrB to inhibit parasite growth was investigated by monitoring the activity of lactate dehydrogenase (pLDH) of *P. falciparum* 3D7 cultured in vitro, as previously described (Makler and Hinrichs 1993; Zininga et al. 2017). The antimalarial compound chloroquine served as a positive control, indicating an IC_50_ of 8.5 nM (Fig. 6). Human GrB inhibited parasite growth in a concentration-dependent fashion, with an IC_50_ of 0.546 µM.

## Discussion

Human GrB is known to interact with the TKD motif of human Hsp70 exposed on the cell surface of tumour cells to facilitate its uptake (Multhoff et al. 2001; Gross et al. 2003; Multhoff 2007). Furthermore, it has been shown that *Plasmodium falciparum*-infected RBCs induce GrB release by NK cells via the expression of host Hsp70 (Böttger et al. 2012). Thus, hHsp70 that is presented on the surface of malaria-infected RBCs is thought to induce the production of GrB by NK cells and facilitate the uptake of GrB by parasite-infected cells which causes their selective death. In addition, hHsp70 parasite-infected RBCs also host the parasite-exported chaperone, PfHsp70-x (Külzer et al. 2012). PfHsp70-x is involved in the trafficking of parasite proteins to the erythrocyte through its association with components of the PTEX translocon (Zhang et al. 2017). Because of this association, we speculated that parasite-infected RBCs may facilitate GrB uptake via PfHsp70-x. Findings of the current study demonstrated that PfHsp70-x binds to GrB in vitro and GrB exhibits antiplasmodial activity. We further showed that PfHsp70-x exhibits a higher affinity for GrB than hHsp70. These findings implicate the involvement of PfHsp70-x in the uptake of human GrB by parasite-infected RBCs. Notably, hHsp70, hHsp60 and hHsp90 were previously reported to be present in the cytosol of iRBCs (Banumathy et al. 2002). On the other hand, PfHsp70-x is known to co-localise with *P. falciparum* Hsp40 co-chaperones in distinct structures called ‘J’ dots (Külzer et al. 2012). In addition, PfHsp70-x occurs in the parasitophorous vacuole and Maurer’s clefts. Since Maurer’s clefts act as membrane cisternae (Külzer et al. 2012), the association of PfHsp70-x with this structure implies a possible role for Pfhsp70-x to influence the constitution of the membrane of parasite iRBCs. This, coupled with our current findings demonstrating that PfHsp70-x exhibits a higher affinity for GrB than hHsp70, suggests that the parasite chaperone mediates GrB uptake by iRBCs more effectively than its human counterpart.

The TKD motif of Hsp70 upregulates the production of GrB by NK cells and facilitates the uptake of GrB into cancer cells (Multhoff et al. 2001; Gross et al. 2003; Multhoff 2007). Our findings show that the TKD motif of PfHsp70-x closely resembles the TKD motif of hHsp70 (Fig. 1a). However, the following substitutions were observed within the TKD motifs of the two proteins: a conserved substitution at position 458R of hHsp70 to 489 K in PfHsp70-x. In addition, hHsp70 is represented by the negatively charged glutamate (E) residue at position 460, while PfHsp70-x is represented by the polar uncharged glutamine (Q) residue at position 491. Perhaps the biggest distinction is the presence of a non-conservative substitution at position 462S (polar, uncharged) of hHsp70 against residue 493E (negatively charged) of PfHsp70-x.

We explored the capability of PfHsp70-x and its TKD motif to bind GrB in vitro. Previously, we demonstrated that the removal of the C-terminal EEVN residues compromises the chaperone function and substrate binding capabilities of PfHsp70-x (Mabate et al. 2018). In the current study, we demonstrated that PfHsp70-x exhibits a higher affinity for GrB than hHsp70. Moreover, the 14-mer TKD motif of PfHsp70-x was found to be sufficient for GrB binding, registering a higher affinity than the hHsp70 TKD motif. We further observed that PfHsp70-x lacking the C-terminal EEVN residues exhibited less affinity for GrB than the full-length protein suggesting that the EEVN motif can augment the affinity of PfHsp70-x for GrB. In addition, our findings suggest that residues 489 K, 491Q or 493E of PfHsp70-x act in isolation or in combination to spur the affinity of PfHsp70-x for GrB.

Furthermore, we explored the role of nucleotides in regulating the interaction of GrB with the two Hsp70s. While PfHsp70-x binding to GrB was nucleotide-independent, ATP appeared to lower the affinity of hHsp70 for GrB. These data suggest that ATP may uniquely regulate hHsp70 binding to GrB. However, the role of nucleotides in regulating GrB recognition by Hsp70 requires further investigation.

Finally, we demonstrated that human GrB exhibits antiplasmodial activity. The findings further suggest that PfHsp70-x augments the uptake of GrB by parasite-infected RBCs. Genes encoding for Hsp70-x occur in malaria parasites of the Laverania subgenus, represented by *P. falciparum* and the chimpanzee parasite, *Plasmodium reichenowi*, but are absent in all other *Plasmodium* species. The beneficial role of PfHsp70-x in GrB uptake could be exploited for future antimalarial therapies against *P. falciparum* which causes the most lethal form of the disease. In addition, the development of GrB antimalarial therapy may provide the possibility to circumvent the growing threat of antimalarial drug resistance.

## Supplementary Information

Below is the link to the electronic supplementary material.

Supplementary file1 (DOCX 8005 KB)
