# Supplementary material for: Human granzyme B binds Plasmodium falciparum Hsp70-x and mediates antiplasmodial activity in vitro
Source: Cell Stress Chaperones. 2023 Apr 19;28(3):321–31. doi: 10.1007/s12192-023-01339-8 (PMC10167072; doi:10.1007/s12192-023-01339-8)
Supplement: Supplementary file 1 — Supplementary file1 (DOCX 8005 KB) [file 12192_2023_1339_MOESM1_ESM.docx]

Supplementary information

**Table S1. SPR equilibrium binding constants for the interaction of GrB with Hsp70 TKD motifs**

| Ligand | Analyte | Nucleotides | Kd/ nM (± SD) | R^2^ |
| --- | --- | --- | --- | --- |
| PfHsp70-xF | GrB | - | 6.46 (± 0.1) | 0.91 |
|  |  | 5 mM ADP | 4.43 (± 0.1) | 0.86 |
|  |  | 5 mM ATP | 9.59 (± 0.2) | 0.89 |
| PfHsp70-xT | GrB | - | 44.00 (± 9,3) | 0.81 |
|  |  | 5 mM ADP | 38.38 (± 8,8) | 0.75 |
|  |  | 5 mM ATP | 51.25 (± 13,6) | 0.70 |
| hHsp70 | GrB | - | 20.79 (± 0.3) | 0.93 |
|  |  | 5 mM ADP | 19.20 (± 0.4) | 0.88 |
|  |  | 5 mM ATP | 441.80 (± 95,3) | 0.95 |
| TKD (PfHsp70-x) | GrB | - | 165.9 (± 29,05) | 9.6 |
| TKD | GrB | - | 349.4 (± 36,80) | 8.8 |
|  |  |  |  |  |

Table legends: *Kd* equilibrium binding constant, SD- standard deviation, R^2^-quantifies the goodness of fit

**Figure S1. Expression and purification of recombinant Hsp70 proteins**

The expression and purification of (**A**) PfHsp70-xF, (**B**) PfHsp70-xT and (**C**) hHsp70 was confirmed using SDS-PAGE and Western blot analyses. The lane M-Page ruler (Thermo Scientific, USA) in kDa; lane C-Total extract for cells transformed with pQE30 plasmid without insert; lane 0-total cell extract transformed with respective construct before IPTG induction; lanes 1, 2, 3, 4, 5 and 24 represent post induction samples collected at 1, 2, 3, 4, 5 and 24 hours respectively. The lane FT represents the flow-through, and lanes W1-W3 -washes and E1 to E4 - elution with 500 mM imidazole.

**Figure S2. Concentration-dependent interaction of PfHsp70-xF/PfHsp70-xT and hHsp70 with GrB**

The representative interaction curves generated from the ELISA representing the association of GrB with either PfHsp70-xF (**A**), PfHsp70-xT (**B**) or hHsp70 (**C**), respectively. The assay was conducted in the presence of varying amounts of GrB towards establishing dose-dependent association. The assay was repeated in presence of 5 mM ATP for PfHsp70-xF-GrB association (**D**), PfHsp70-xT-GrB association (**E**) and hHsp70-GrB (**F**); and in the presence of 5 mM ADP for PfHsp70-xF-GrB association (**G**), PfHsp70-xT-GrB association (**H**) and hHsp70-GrB (**I**), respectively. Curves representing interaction of GrB with TKD peptide of PfHsp70-x (**J**) or hHsp70 (**K**) are shown. The error bars represent the SDs obtained about the means of at least three assays conducted using independently purified recombinant protein batches.
